# Young Adults With Higher Motives and Expectancies of Regular Cannabis Use Show Poorer Psychosocial Functioning

**DOI:** 10.3389/fpsyt.2020.599365

**Published:** 2020-12-15

**Authors:** Danielle Amiet, George J. Youssef, Lauryn J. Hagg, Valentina Lorenzetti, Linden Parkes, Nadia Solowij, Murat Yücel

**Affiliations:** ^1^BrainPark, School of Psychological Sciences and Monash Biomedical Imaging Facility, Turner Institute for Brain and Mental Health, Monash University, Melbourne, VIC, Australia; ^2^Centre for Social and Early Emotional Development, School of Psychology, Deakin University, Geelong, VIC, Australia; ^3^Centre for Adolescent Health, Murdoch Children's Research Institute, Royal Children's Hospital, Melbourne, VIC, Australia; ^4^Neuroscience of Addiction & Mental Health Program, Healthy Brain and Mind Research Centre, Faculty of Health Sciences, School of Behavioural & Health Sciences, Australian Catholic University, Melbourne, VIC, Australia; ^5^Department of Bioengineering, School of Engineering & Applied Science, University of Pennsylvania, Philadelphia, PA, United States; ^6^School of Psychology and Illawarra Health and Medical Research Institute, University of Wollongong, Wollongong, NSW, Australia; ^7^The Australian Centre for Cannabinoid Clinical and Research Excellence, New Lambton Heights, NSW, Australia

**Keywords:** cannabis (marijuana), latent class, regular users, psychosocial functioning, young adult, motives, expectancies

## Abstract

**Background:** Young adults regularly using cannabis represent a uniquely vulnerable yet heterogeneous cohort. Few studies have examined user profiles using cannabis use motives and expectations. The association between user profiles and psychosocial functioning among only regular users remains unexplored. This exploration is important to improve public education efforts and design tailor treatment approaches.

**Methods:** Regular cannabis users (at least weekly; *n* = 329) completed an online survey via Amazon Mechanical Turk. The survey measured levels of cannabis use, other substance use, motives and expectations of cannabis use, symptoms of psychosis, depression, anxiety and stress, and reckless behavior such as getting high before work or driving under the influence of cannabis. Latent class analysis was performed using motives and expectations to identify data driven patterns of regular cannabis use. Classes were then used to investigate mental health and behavioral correlates of differences in motives and expectations.

**Results:** A 2-class solution provided the best fit to the data; Class 1: Low Motives and Expectancies (*n* = 158) characterized by lower endorsement across all motivation and expectation variables, and Class 2: High Motives and Expectancies (*n* = 171) characterized by endorsing multiple motivations, and higher positive and negative expectations of cannabis use. Classes differed in a range of cannabis use variables; e.g., greater proportion of peer use in Class 2. The High Motives and Expectancies users reported higher symptoms of psychosis (positive and negative symptoms), depression, anxiety, and stress. A higher proportion met the criteria for a cannabis use disorder compared with Low Motives and Expectancies users. High Motives and Expectancies users reported higher mean problems with nicotine dependence and illicit drug use other than cannabis and were more likely to get high before work and drive under the influence of cannabis.

**Conclusions:** There is heterogeneity among young regular cannabis users in their motivations and expectancies of use and associated psychosocial functioning. Understanding motives and expectancies can help segregate which users are at higher risk of worse functioning. These findings are timely when designing targeted assessment and treatment strategies, particularly as cannabis is further decriminalized and accessibility increases.

## Introduction

Cannabis, also known as marijuana, is the most widely consumed illicit substance worldwide, particularly among young adults (1). Young adults with cannabis abuse or dependence represent 7.5% of the total population and 62.5% of all those with cannabis use disorders (2). Increasingly, cannabis is being decriminalized for medicinal and recreational purposes across the globe, including some states in the United States (US). In the US states which have legalized cannabis, the price has decreased making cannabis more accessible. Likewise, the potency of cannabis products has increased, which has been linked to poorer psychosocial functioning (3, 4). One report suggests that potency, determined by the percentage of Δ^9^-tetrahydrocannabinol (THC) responsible for the “high” that users experience, has increased from 9 to 30% in the past three decades (5). Whilst laws that support the legalization of cannabis try to achieve social justice aims (e.g., reducing the prison population) and generate tax revenue, cannabis-related psychosocial harms are also at risk to increase contributing to a greater burden of disease, such as increased hospital admissions, and higher social and economic damage (6, 7).

Cannabis use typically begins in adolescence and peaks in young adulthood (8). The prevalence of usage has increased in both age groups (9, 10), however is highest amongst young adults. This is concerning given that the perception of harm associated with regular use in this cohort has been decreasing over time (11). Young adults report the highest reluctance to seek treatment for cannabis-related problems compared to any other age group, therefore hindering their opportunity for recovery from psychosocial harm (12). As such, young adults represent a uniquely vulnerable group, as exposure to cannabis can result in harmful consequences for their mental health, employment and education, and increased risk of driving related accidents and fatalities (13–16). As such, research that focuses solely on young adults will help improve public education efforts and the design of more tailored treatment strategies.

Regular cannabis use is typically the strongest predictor of later psychosocial impairment (13), next to potency (17, 18) and age of onset (10). Despite this, not all regular users, hereby defined as at least weekly consumption, report poor psychosocial functioning (19–21), with evidence suggesting the proportion is only one third of regular users (22). Psychosocial dysfunction experienced by regular users includes increased symptoms of psychosis (23), apathy (24), depression and anxiety (25), poor employment and educational outcomes (26), and increased risk of motor vehicle crashes (27). Despite clear, documented harms associated with the regular consumption of cannabis, it is unclear how to disentangle which regular users are at higher risk. Understanding the features which segregate regular users is necessary to inform prevention and treatment strategies targeting young adult users.

Previous research investigating cannabis-related harms has almost always focused on comparing groups by their frequency of use, for example comparing daily users, occasional users (e.g., monthly), and abstainers (28–30). Yet no study, to the authors' knowledge, has examined how psychosocial functioning varies in exclusively regular cannabis users. A focused approach to understanding subgroups of regular cannabis users is required in order to identify the nuanced differences in regular user profiles and how this relates to subsequent functioning. Research which groups together regular users and compares them against occasional users and abstainers does not create clinically meaningful and tailored intervention strategies for the diverse regular users that seek treatment (31). In addition, not all regular users meet criteria for a cannabis use disorder (CUD), which suggests that further investigation is required to understand exactly how regular users differ from one another (32). One means of classifying subtypes of regular users, that does not rely on comparison according to frequency of use, is through exploring subjective experiences, specifically a person's motivations for use and any positive or negative consequences they expect from using cannabis.

The association between motivations and expected outcomes for cannabis use, and adverse psychosocial functioning, has received growing attention. Emerging evidence shows that the motivation for using cannabis can separate problem and non-problem users (33). There are several motives commonly referred to within the literature, which include coping (e.g., to forget problems), enhancement (e.g., pleasant feelings), social (e.g., improves parties), conformity (e.g., fitting in), expansion (e.g., increasing creativity), and routine (e.g., boredom). One study found social, enhancement and coping motives were associated with higher consumption (34), whilst another study found that cannabis dependent users highly endorsed every motivation for their use, and cannabis abusers only endorsed enhancement and expansion motives (35–39). Various studies have pointed to coping-related motivations as the most robust predictor of worse psychosocial functioning (40).

Another mechanism to disentangle the vast differences in psychosocial functioning between regular users is by examining positive and negative expectations of cannabis use. One study found that negative expectancies (e.g., being confused) were associated with greater cannabis dependency, while positive expectancies (e.g., feeling more outgoing) were associated with greater weekly consumption. Coupled together, both high positive and negative expectancies were linked to impaired psychological functioning, such as depression and anxiety (41). Another study found that positive expectancies, but not negative expectancies, were associated with worse mental health outcomes and problems such as missing school or work (42). Despite evidence supporting the role of subjective experiences in explaining varying patterns of psychosocial functioning, motivations, and expectations are yet to be collectively investigated in a cohort of only young adult regular users.

One of the difficulties associated with examining motives or expectancies around cannabis use is that any one user may endorse multiple motivations or outcomes from cannabis use. Consequently, an individual's personal pattern of endorsement across these broad motives and expectancies may be more relevant to explaining the heterogenous outcomes of regular users than focusing on any one variable in isolation. Latent class analysis (LCA) is an analytical tool that permits such an examination by identifying subgroups within a heterogeneous sample who share a similar pattern of endorsement across multiple items (43, 44). Many studies in recent years have used LCA to identify subtypes of cannabis users (45–49), including some who have looked at motivations and expectations (8). Studies examining motives and expectancies have found endorsing multiple motivations and negative expectations is associated with poorer functioning. However, none of these studies have estimated such models within exclusively regular cannabis users. As such, class formation in these samples will have been heavily influenced by the frequency of use and thus a refined understanding of the motives and expectancies within an exclusively regular using sample will have been diluted.

A comprehensive approach is needed to disentangle the characteristics associated with varied psychosocial functioning in regular cannabis users, particularly during young adulthood where life-long behavioral patterns are established, including continuation of regular cannabis use (6). This research is timely given the recent trends toward decriminalizing cannabis products in several international jurisdictions which has seen an increase in cannabis-related hospital admissions (3, 4). An increase in dependent users, including young adults, is also likely as more states move toward legalization for both medicinal and recreational purposes, and the availability of cheaper, and more potent products enter the market (50). As such, there is a need to develop an improved understanding of factors that predict individuals who go on to problematic patterns of use. This study aimed to characterize common motivation and outcome expectancy patterns in a sample of exclusively regular cannabis users. Whilst we had no a *priori* hypotheses regarding the number of LCA subgroups that would be identified, we expected to find at least one latent class of cannabis users with a higher endorsement of coping motives, and one latent class of users with higher positive expectations. Once identified, we then aimed to characterize the psychosocial functioning of each class across a range of outcomes such as psychopathology, education and employment, and engagement in reckless behaviors.

## Materials and Methods

### Participants and Procedure

Regular cannabis users (*n* = 329) from the United States were recruited in August 2015 via Amazon Mechanical Turk (MTurk). Inclusion criteria included: (1) 18–30 years old; (2) cannabis use at least weekly for the past 12 months; (3) fluent English; (4) no other drug use more than once a month in the past 12 months; and (5) no diagnosis or treatment of problematic alcohol and drug use besides cannabis. Only eligible participants were reimbursed US$4.50 for their time, which was consistent with the recommended hourly rate at the time of data collection. Written informed consent was provided prior to participation. Ethical approval was granted from the Monash University Human Research Ethics Committee (CF15/1235–2015000576).

Of participants deemed eligible to continue, 357 completed the questionnaire. Despite past research indicating attention levels are similar across MTurk, convenience sampling and high-quality sampling methods (51), we embedded validity item checks designed to test if the participant was paying attention to further increase the quality of data collected. Only 28 participants were further excluded and not reimbursed for failing to correctly answer at least 70% of the embedded validity item checks (i.e., >14/20), leaving 329 participants for analysis (52, 53).

### Measures

#### Indicators Used in LCA

The 24-item Extended Marijuana Motives Measure (Extended-MMM) examines different motivations for using cannabis via six subscales: Coping (e.g., “To forget my worries”), Enhancement (e.g., “Because I like the feeling”), Social (e.g., “To be sociable”), Conformity (e.g., “To be liked”), Expansion (e.g., “To know myself better”), and Routine (e.g., “Out of boredom”). The scale demonstrates adequate internal reliability (Cronbach's α 0.68–0.85), factorial validity, and criterion-related validity (54). Items were measured using a 5-point Likert scale (1 = *Almost Never*, 5 = *Almost Always*), with higher scores indicating a greater endorsement of each motivation (40).

The 45-item Cannabis Expectancy Questionnaire (CEQ) measures anticipated consequences from using cannabis via two subscales: Positive Cannabis Expectancy (e.g., “Smoking cannabis makes me happy”) and Negative Cannabis Expectancy (e.g., “Smoking cannabis makes me confused”). Both subscales demonstrate high internal consistency (α = 0.89–0.93) and established criterion validity across two samples. Items were measured using a 5-point Likert scale (1 = *Strongly Disagree*, 5 = *Strongly Agree*). Higher scores indicate an increased positive or negative expected outcome from cannabis use (55–57). The total scores from the Extended-MMM and CEQ subscales served as the continuous indicators in the LCA analysis.

#### Measures of Cannabis Use

Levels of cannabis use were measured across various domains. See Table 1 for the items written to measure levels of cannabis use.

**Table 1:** Items asked to measure levels of cannabis use.

**Questions**	**Available options**
What age were you when you tried cannabis/marijuana for the first time?	10–30 years old
What age were you when you started to use cannabis/marijuana regularly?	10–30 years old
When do you usually use cannabis?	All day (yes or no)
With whom do you usually use cannabis?	Alone OR Friends/partner OR Family OR Others
Where do you usually use cannabis?	In public OR At home OR At friend's house
Which of the following do you usually use at the same time as using Marijuana/Cannabis?	Alcohol OR Other drugs
About what proportion of your friends and acquaintances currently use Marijuana/Cannabis?	Few/None OR Half or more
When using marijuana, what type do most commonly use?	Mostly dried heads OR Mostly dried leaves OR Sinsemilla OR I don't know
Which route of administration do you usually use?	Joint OR Pipe OR Water Pipe/Bong OR Blunt OR Vaporizer OR Other
On a scale from 1 to 10 (where 1 = sober, 5 = stoned, 10 = very blazed), how high do you usually get?	1–10

#### Measures of Psychosocial Functioning

The 42-item Community Assessment of Psychic Experience (CAPE) measures positive psychotic experiences (20-items), negative psychotic experiences (14-items), and depressive symptoms (8-items). The scales demonstrate good stability, reliability (α = 0.81–0.83) and discriminant validity. Participants rated both frequency and distress of symptoms on a 4-point Likert scale. Higher scores indicate greater levels of psychotic-like symptoms, with a cut-off score of 50 on the frequency dimension of the positive subscale indicating a possible psychotic disorder (58, 59). In the analyses, only the frequency measure of the positive and negative psychotic experience subscales was used.

The 21-item Depression Anxiety Stress Scale (DASS-21) measures symptoms of depression, anxiety and stress. The scale demonstrates good reliability (α = 0.87–0.94) and concurrent validity. Items were measured on a 4-point Likert scale (0 = *Never*, 3 = *Almost always*), with higher scores indicating greater symptom levels. Cut-off scores above 4 for depression, 3 for anxiety and 7 for stress indicate above normal symptoms (60, 61).

The 18-item Apathy Evaluation Scale was used to measure levels of apathy. Good reliability (α = 0.94) and validity of this scale have been previously established (62). Items were measured on a 3-point Likert scale (1 = *Not at all*, 3 = *Somewhat a lot*). Scores ranged from 18 to 72, with scores above 38 indicating apathy (63).

The 16-item Cannabis Use Problems Identification Test (CUPIT) is a self-report measure used to detect problematic cannabis use. It has two subscales, “Impaired Control” and “Problems.” The scale demonstrates high internal reliability (α = 0.83–0.92) and good construct, discriminative, diagnostic and predictive validity. Items were measured on different Likert scales (e.g., 1 = *Never*, 5 = *Always/All the time*). Higher scores indicate a higher likelihood of cannabis-induced problems and dependence. A total cut-off score of 12 indicates risk of developing a CUD, whilst 20 indicates meeting criteria for a CUD. The criteria for diagnosis are in-line with the Diagnostic Statistical Manual, 4th edition (DSM-IV) and the International Classification of Diseases, 10th Edition [ICD-10 (64)].

An additional *ad hoc* item was written to further measure psychological dysfunction. It stated: “Have you ever sought treatment for issues surrounding mental illness (e.g., depression, anxiety, psychosis, etc.)?” which was scored as either “Yes,” or “Never.”

To measure reckless behavior, we asked two questions: (1) “Do you ever drive whilst stoned/high?” which was scored as either “Rarely/Never” or “Sometimes/Always,” and (2) “Do you ever use cannabis/get high just before or during work?” which was scored as either “Never/Rarely” or “Sometimes/Often.”

#### Covariate Adjustment Variables

Analyses were adjusted for the following demographic variables: age, gender, education, employment, Caucasian ethnicity, problematic illicit drug use (other than cannabis), nicotine dependence, and alcohol-related problems. The alcohol and other drug use measures are listed below.

The 10-item Drug Abuse Screening Test (DAST) measures illicit substance abuse using a dichotomous “Yes” or “No” response format (65). Questions were adapted to measure lifetime use rather than for the previous 12 months. Higher scores indicate increased risk of harm from illicit drug use. The scale was categorized using three cut-off points: scores of 0 indicated “low” risk of previous illicit drug problems; scores of 1–2 indicated “moderate” risk; and scores of 3 and above indicated “high” risk. The scale demonstrates high internal consistency (α = 0.86–0.94) and good criterion validity (66, 67).

The 6-item Fagerström Test for Nicotine Dependence (FTND) measures level of dependence on nicotine. Higher scores indicate a higher level of nicotine dependence. The FTND was categorized using three cut-off points: scores of 0–2 indicated “low” risk of nicotine dependence; scores of 3–4 indicated “low/moderate” risk; and scores of 5 and above indicated “moderate/high” risk. The scale demonstrates moderate internal consistency (α = 0.72–0.74) and good convergent and discriminant validity (68).

The 10-item Alcohol Use Disorders Identification Test (AUDIT) measures alcohol dependence and specific consequences of harmful drinking. Higher scores indicate more hazardous alcohol consumption. The scale was categorized using three cut-off points: scores of 0–7 indicated “low” risk of hazardous drinking and related problems; scores of 8–15 indicated “moderate” risk; and scores of 16 and above indicated high risk.

### Statistical Analysis

Latent class analysis (LCA) was used to classify regular cannabis-users into subtypes based on their responses across the coping, enhancement, social, conformity, expansion, and routine motives subscales, and positive and negative expectancies subscales. Specifically, a series of LCA models (from 2 to 6 classes) were performed using Mplus [version 7.2 (69, 70)]. All indicator variables were *z*-score standardized prior to LCA to assist interpretability. The optimal number of latent classes was identified based on low Akaike Information Criterion (AIC) and Bayesian Information Criterion (BIC) values (71, 72), and the Vuong–Lo–Mendell–Rubin (VLMR) and Lo–Mendell–Rubin (LMR) adjusted likelihood ratio tests which provide a *p*-value comparing the fit of a model with k-classes to a null hypothesis model comprising k-1 classes (69). Entropy values, which indicate the degree of homogeneity within, and independence between, each class was also used to characterize the classes (73), but as recommended by others (74) was not used to determine the optimal number of classes. Entropy values >0.80 suggest a strong probability that an individual belongs to the class for which they have the highest probability of membership (i.e., “most likely class membership”), and thus this most likely class variable can be used as an observed variable in subsequent regression analysis (75).

Once the best fitting LCA model was identified, we estimated a series of regression models where we regressed the outcomes of interest on a categorical variable denoting the participants' most likely class membership. The correlates were broadly classed as demographic factors (i.e., gender, marital status, income, age), cannabis use factors (e.g., preferred route of administration), and psychological and substance use factors. All analyses were adjusted for age, gender, education, employment, Caucasian ethnicity, and alcohol-related problems, nicotine dependence and problematic illicit drug use other than cannabis. Specific covariates were removed if they were used as the dependent variable (e.g., when the AUDIT total score was measured, AUDIT was removed as a covariate). Missing data in the criterion variable ranged from 0 to 19.5% (e.g., the latter for “Route of administration”) and were handled using Full Information Maximum Likelihood (FIML) estimation (51). There were no missing data in any of the predictor or covariate variables.

## Results

### Demographic Information

The mean reported age was 25.95 (SD = 3.29) years. The sample comprised 133 females and 196 males representing an approximate 3:2 ratio in favor of males, consistent with prevalence rates of regular cannabis users in other Western nations (34). Overall, most participants identified as Caucasian (77%) and in a relationship (44%), with roughly a quarter living alone (26%). Three-quarters (75%) had completed, or were completing, either university or trade school. Most participants were employed (78%). There were no significant differences between genders on demographic factors, except that more females (60%) endorsed being in a relationship than males (32%).

### Class Solution

Table 2 presents the fit statistics for 2- through to 6-class latent class models. The VLMR and LMR suggested that a 2-class model was significantly better fitting than a 1-class model, while there was only weak evidence to suggest a 3-class model was better than a 2-class model. The AIC and BIC values were found to continue to increase across the models, with models with more than 6 classes not estimable or had class sizes that were impractically small. Given the AIC and BIC did not reach a low point, we used the LMR and VLMR results and retained a 2-class model (entropy = 0.78) for further analysis. Given that there was some weak evidence for a 3-class model, we also conducted all subsequent analyses using the 3-class model but provide this as Supplementary Material for the interested reader. Where relevant, we compare the results of the 2- and 3-class models in text.

**Table 2:** Fit indices for the estimated latent class models (*n* = 329).

	**2-class model**	**3-class model**	**4-class model**	**5-class model**	**6-class model**
AIC	7022.7	6827.764	6684.699	6618.802	6507.826
BIC	7117.601	6956.83	6847.93	6816.197	6739.385
Entropy	0.78	0.853	0.879	0.83	0.879
VLMR (*p*-value)	0.0003	0.0601	0.0651	0.3988	0.5657
LMR (*p*-value)	0.0004	0.0625	0.068	0.4038	0.5716

*AIC, Akaike Information Criteria; BIC, Bayesian Information Criterion; VLMR, Vuong-Lo-Mendell-Rubin Likelihood Ratio Test; LMR, Lo-Mendell-Rubin Adjusted LRT Test; p-value testing the null hypothesis that a model with one less class has better fit*.

### 2-Class Model

**Figure 1:**
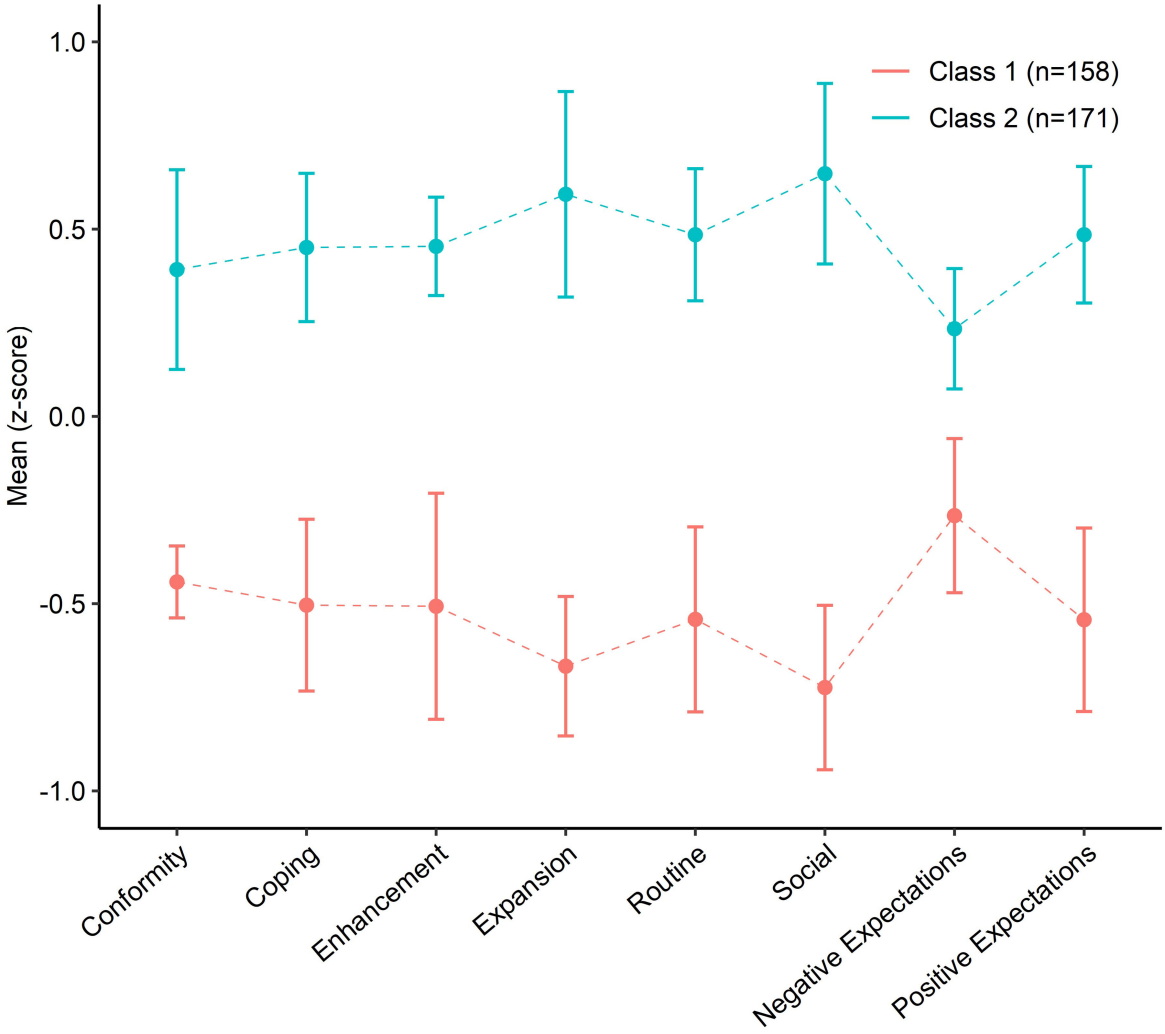
Latent profile of participants based on marijuana use motives and cannabis use expectancies. Error bars represent 95% confidence intervals.

The 2-class model features (seen in Figure 1) were largely differentiated by magnitude differences across the LCA indicators. Class 1 was named Low Motives and Expectancies (48% of the sample) and Class 2 named High Motives and Expectancies (52% of the sample). The High Motives and Expectancies class was higher on all Extended-MMM factors and reported higher negative and positive expectations from cannabis use, compared with Low Motives and Expectancies class. The magnitude of differences across variables was large and ranged from Cohen's d = 0.50 (Negative Expectancies) to d = 1.40 (Social Motives). For comparison, the classes found in the 3-class model (see Supplementary Material) were largely consistent with the 2-class model. Specifically, Class 1 in the 3-class model was similar to Class 1 of the 2-class model, comprised similar low motives and expectancies, with Class 2 of the 2-class model appearing to be split into two separate groups. The latter two groups differed marginally on variables such as negative expectations and social motives, however the most discriminating factor was the Conformity motivation variable. Given the consistency in classes, we continue to present the more parsimonious 2-class solution in subsequent analyses.

### Correlates of Class Membership

The 2-class model was the most effective in segregating regular users, based on their motives and expectancies of cannabis use. We examined whether the classes were associated with a range of psychosocial correlates, inspecting the marginal mean differences between Class 1 (Low Motives and Expectancies) and Class 2 (High Motives and Expectancies) across demographic, cannabis use, mental health ,and substance use factors. Table 3 highlights demographic variables. Class 2 were more likely to be employed and have a higher mean age, although the difference in age was negligible (~1 year).

**Table 3:** Means and confidence intervals of demographic outcome variables (2-class model)^a^.

**Variable**	**Low motives and expectancies (*n* = 158) *M* (95% CI)**	**High motives and expectancies (*n* = 171)*M* (95% CI)**	**Significant contrasts**
Age	26.48 (26, 26.97)	25.45 (24.98, 25.92)	C1>C2^**^
Percentage of males	56% (49%, 63%)	63% (56%, 70%)	
Percentage in a relationship	47% (41%, 54%)	41% (34%, 47%)	
Percentage who have completed secondary/high school	78% (72%, 85%)	74% (67%, 80%)	
Percentage who are currently employed	72% (65%, 78%)	84% (78%, 89%)	C1 < C2^*^
Percentage who have Caucasian ethnicity	78% (71%, 84%)	75% (69%, 82%)	
Percentage who live alone	28% (22%, 35%)	25% (19%, 30%)	

* *p < 0.05*,

** *p < 0.01*.

a *Analyses were adjusted for age, gender, education, employment, Caucasian ethnicity, and total scores of the AUDIT, DAST and FTND*.

**Table 4:** Means and confidence intervals of cannabis use variables (2-class model)^a^.

**Variable**	**Low motives and expectancies (*n* = 158) *M* (95% CI)**	**High motives and expectancies (*n* = 171)*M* (95% CI)**	**Significant contrasts**
Age of first use	16.94 (16.51, 17.36)	16.30 (15.9, 16.71)	C1>C2^*^
Age of regular use	20.40 (19.93, 20.88)	19.14 (18.68, 19.59)	C1>C2^***^
Self-reported “high” during use (10 = very blazed)	5.50 (5.22, 5.78)	6.40 (6.13, 6.68)	C1 < C2^***^
Percentage who use cannabis all day	7% (3%, 11%)	27% (20%, 34%)	C1 < C2^***^
Percentage with half or more peers using cannabis	44% (37%, 52%)	74% (67%, 81%)	C1 < C2^***^
Percentage who sometimes/always drive high	18% (12%, 24%)	34% (27%, 41%)	C1 < C2^**^
Percentage who sometimes/often go to work high	13% (7%, 18%)	32% (25%,39%)	C1 < C2^***^
Percentage usually using cannabis and alcohol	20% (14%, 26%)	17% (12%, 23%)	
Number of days using cannabis per week			
1–2 times	0.46 (0.39, 0.54)	0.25 (0.19, 0.32)	C1>C2^***^
3–5 times	0.30 (0.23, 0.38)	0.28 (0.22, 0.35)	
6–7 times	0.23 (0.17, 0.3)	0.47 (0.39, 0.54)	C1 < C2^***^

* *p < 0.05*,

** *p < 0.01*,

*** *p < 0.001*.

a *Analyses were adjusted for age, gender, education, employment, Caucasian ethnicity, and total scores of the AUDIT, DAST, and FTND*.

Table 4 highlights the differences between classes on levels of cannabis use. Class 2 had an earlier age of first use and regular use and reported higher scores when asked “how high” they like to get on a scale of 1–10. Class 2 also had higher percentages of users who use cannabis all day and who have half or more of their peers using cannabis and were more likely to get high just before or during work and drive while under the influence of cannabis. Class 2 were more likely to use cannabis near daily, while Class 1 were more likely to use only 1–2 times per week. In addition, Class 2 had a higher percentage who preferred using with friends/partners (89%, *p* < 0.01) and family members (19%, *p* < 0.01) compared to Class 1 (76 and 8%, respectively). Class 2 were more likely to engage in cannabis use in public places (45%, *p* < 0.05), at a friend's house (81%, *p* < 0.01), or within their own home (96%, *p* < 0.05) compared to Class 1 (32, 66, 86%, respectively). There were no differences between classes on their preference to use alone, preferred route of administration, or preferred type of marijuana except for sinsemilla which was more highly endorsed by Class 2.

Table 5 highlights the differences between classes across a range of mental health and substance use variables. Class 2 had higher symptoms of psychosis (positive and negative symptoms), depression, anxiety, and stress compared with Class 1. Class 2 also reported higher problematic cannabis use and were flagged as more likely to meet the criteria of a CUD than Class 1. Class 1 reported lower mean problems with nicotine dependence and drug use other than cannabis compared to Class 2.

**Table 5:** Means and confidence intervals of mental health and substance use outcome variables (2-class model)^a^.

**Variable**	**Low motives and expectancies (*n* = 158) *M* (95% CI)**	**High motives and expectancies (*n* = 171)*M* (95% CI)**	**Significant contrasts**
**Mental health outcomes:**			
Total Apathy Evaluation Scale score	42.61 (41.55, 43.67)	42.4 (41.39, 43.42)	
CAPE Positive Psychotic Experiences subscale	24.82 (23.8, 25.83)	27.83 (26.85, 28.81)	C1 < C2^***^
CAPE Negative Psychotic Experiences subscale	21.23 (20.11, 22.34)	23.27 (22.2, 24.34)	C1 < C2^*^
DASS-21 Depression subscale	2.71 (2.09, 3.33)	3.99 (3.4, 4.58)	C1 < C2^**^
DASS-21 Anxiety subscale	1.9 (1.43, 2.36)	3.34 (2.89, 3.78)	C1 < C2^***^
DASS-21 Stress subscale	2.83 (2.3, 3.37)	4.62 (4.11, 5.13)	C1 < C2^***^
Percentage who have ever sought mental health treatment	15% (10%, 21%)	17% (12%, 22%)	
**Problematic cannabis use:**			
Total CUPIT score	25.29 (23.73, 26.84)	33.33 (31.83, 34.82)	C1 < C2^***^
CUPIT Impaired Control subscale	22.86 (21.53, 24.19)	29.74 (28.46, 31.01)	C1 < C2^***^
CUPIT Problems subscale	2.42 (1.96, 2.89)	3.59 (3.14, 4.04)	C1 < C2^**^
CUPIT Cut-off score ≥12	95% (92%, 99%)	99% (98%, 100%)	
CUPIT Cut-off score ≥20	74% (66%, 81%)	96% (94%, 99%)	C1 < C2^***^
**Substance use outcomes:**			
Total AUDIT score	11.4 (10.41, 12.39)	12.62 (11.67, 13.57)	
Total DAST score	1.05 (0.85, 1.26)	1.45 (1.26, 1.65)	C1 < C2^**^
Total FTND score	0.78 (0.46, 1.1)	1.29 (0.98, 1.6)	C1 < C2^*^

* *p < 0.05*,

** *p < 0.01*,

*** *p < 0.001*.

a *Analyses were adjusted for age, gender, education, employment, Caucasian ethnicity, and total scores of the AUDIT, DAST, and FTND*.

Notably, when conducting these analyses with the 3-class model, there were little differences in interpretation since the pattern of results comprised Class 1 being different from both Class 2 and 3, but few meaningful effects differentiating Class 2 and 3. The only variables found to differentiate Class 2 and 3 were positive psychosis symptoms, anxiety symptoms, and the CUPIT Problems subscale score, which were all higher in Class 3 than Class 2.

## Discussion

This is the first study to examine the profiles of exclusively regular cannabis users during young adulthood, using latent class analysis. In particular, this study focused on users' motivation and their expected outcomes of cannabis use as the basis of each profile. The LCA model in this study identified two different classes of regular cannabis users: Class 1 *Low Motives and Expectancies*, and Class 2 *High Motives and Expectancies*. As expected, one latent class (i.e., Class 2) had higher positive expectancies for using cannabis. Interestingly, coping did not emerge as a sole discriminating factor in either the 2- or 3-class model, despite past research suggesting that coping was one of the most robust motivational predictors of poorer outcomes. Instead, our study found that the class who experienced the worst psychosocial impairment (i.e., Class 2) reported higher scores across all motivational indicators, which was found in only one other study by Bonn-Miller et al. (34). It is worth noting that whilst Bonn-Miller et al. examined current users of varying frequencies, the majority used at least weekly, suggesting that perhaps among regular users, coping is less of a discriminating motivator. Overall, Class 2 represented 52% of the sample, which is in line with past research demonstrating heterogeneity in the psychosocial functioning, mental health and behavioral outcomes of regular users. We note all regression analyses controlled for demographic variables, problematic alcohol use, nicotine dependence, and other drug use.

Our findings suggest that the motivations and expected outcomes of cannabis use are associated with patterns of use. In line with past research on risk factors for poorer functioning, Class 2 were more likely to be near daily users, whilst Class 1 were more likely to use cannabis 1–2 times per week. Likewise, Class 2 were more likely to prefer sinsemilla, a more potent cannabis variety, and had a lower mean age of first use and regular use, compared with Class 1. A novel contribution to the literature was assessing patterns of use among regular users beyond simple frequency or mode of cannabis use. Specifically, the High Motives and Expectancies users (Class 2) had a higher mean self-reported “high” when using cannabis, were more likely to have half or more of their peers use cannabis, and were more likely to use cannabis all day compared to the Low Motives and Expectancies users (Class 1). Likewise, Class 2 had a higher percentage who preferred using with friends, partners, and family, and use cannabis in public, at a friend's house, or at home compared to Class 1. These findings support the notion of heterogeneous use patterns even among regular users.

Across mental health indicators, the High Motives and Expectancies users had significantly higher symptoms of positive and negative psychotic experiences, depression, anxiety, and stress compared with the Low Motives and Expectancies users. This supports past research which has shown that endorsing multiple motivations for using cannabis (40, 41) and having higher positive expectations of cannabis use (27) is associated with worse mental health outcomes. However, with a low prevalence of mental health symptoms across groups, and only Class 2 exceeding the cut-off score of above normal anxiety symptoms, this finding should be interpreted with caution. The only mental health outcomes that Class 1 and Class 2 did not differ on was apathy levels and whether they had ever sought treatment for mental health issues. That said, it is worth noting both groups scored a mean above 38 indicating they were both clinically apathetic.

When compared on patterns of substance use, the High Motives and Expectancies (i.e., Class 2) users showed worse functioning. On the CUPIT, Class 2 scored significantly higher than Class 1 on the total score and both subscales, indicating worse problematic cannabis use and impaired control. Interestingly, whilst not significantly different, the percentage of users who scored above the cut-off score to indicate risky cannabis use was very high at 95 and 99% for Class 1 and 2, respectively. Whilst there was a significant difference between Class 1 and 2 regarding an indication of a CUD, the vast majority of both classes still exceeded the cut-off score with 74 and 96%, respectively. Given the relatively low prevalence of mental health symptoms highlighted earlier, and the high percentage of users exceeding cut-off scores above what past research indicates is prevalent within regular users, our findings suggest the CUPIT may not be sensitive enough to distinguish between problematic and non-problematic cohorts who already endorse using cannabis regularly. In addition, Class 2 had a higher nicotine dependence and higher abuse of illicit drugs other than cannabis compared with Class 1, but there were no differences on problematic alcohol use.

Across other psychosocial indicators, interestingly, Class 2 had a higher percentage of users who were currently employed compared with Class 1. However, this study did not distinguish between secure vs. insecure forms of current employment, so it is difficult to ascertain whether this indicates better or worse functioning. In contrast, Class 2 were more likely to engage in reckless behavior such as attending work whilst high on cannabis or driving under the influence of cannabis. This is concerning given that acute cannabis consumption increases the risk of motor vehicle crashes and fatalities (16), decreases workplace performance and increases absenteeism (11). The public health and economic implications for understanding which patterns of regular use are associated with increased reckless behavior is important for improving public awareness campaigns and tailoring treatment regimes.

The implications of this study are 2-fold. From a clinical perspective, our results highlight the importance of better understanding users' motivations and expectations of cannabis use in addition to the standard objective measures of frequency, potency, and age of onset. Young adults consume the highest quantity of cannabis compared to other age groups and are the least likely to seek treatment for cannabis-related problems (7, 33), which is why targeted intervention and prevention strategies are required to minimize impaired functioning later in life (32). As demonstrated by this study, and as supported by past research, there is large heterogeneity between subtypes of regular users (13, 37). Our results not only found that one class of regular users had higher motives and expectancies, each class significantly differed across a range of cannabis use variables such as their preference to use cannabis all day or the percentage of peers they associate with who also use cannabis. These additional comparisons were made to further disentangle the different subtypes of regular cannabis users and aid the creation of tailored treatment strategies. Implementing a “one-size-fits-all” approach to the assessment and treatment of psychosocial impairment will likely have limited success, particularly if the focus is largely on asking about regular use or administering questionnaires such as the CUPIT in isolation (31, 40). Comprehensive and tailored approaches toward assessing and treating cannabis use problems for young adults, particularly those which recognize the nuanced differences in regular users, are needed to reduce associated impairment.

Second, there are public health and policy implications, particularly given the large proportion of young adults who are open about using cannabis regularly yet have a low perception of harm associated with this drug (11). The results of our study show that regular users who are highly motivated and experience higher positive and negative expectations associated with cannabis use have poorer psychosocial functioning. However, as this study did not investigate causality, it is possible that the reverse is also true, and that the onset of psychosocial dysfunction preceded the onset of regular cannabis use. Nevertheless, these findings aim to improve public education efforts targeting regular cannabis users during and even before young adulthood about the association between motivations and expectations for cannabis use and mental health, substance use, and behavioral outcomes. Improving education about the associated risks will allow young adults to make more informed decisions about cannabis. For jurisdictions looking to decriminalize use, and for those where cannabis is already legal, early intervention, and education about the risks of being highly motivated and expecting positive outcomes from cannabis use is key to decreasing associated mental health issues, cannabis dependency, reduced safety and productivity in the workplace, and increased motor vehicle crashes and fatalities. Whilst it is not inevitable that the legalization of recreational cannabis use will result in increased psychosocial impairment, the largely unregulated potency of cannabis, increased availability and decrease in costs are not encouraging. Our recommendations support the growing literature encouraging governments to use part of their tax revenue to monitor the long-term negative consequences of cannabis use in order to minimize the associated social and economic costs and burden of disease (3–10).

This research is not without limitations. First, the cross-sectional design prevents the inference of causality. However, longitudinal research shows cannabis use usually precedes the onset of psychosocial dysfunction in young adults, and not the reverse, and that baseline characteristics such as motives can predict later psychosocial dysfunction (13, 37). Second, the reliance on self-report measures potentially biases the results. This can result from memory recall issues, common in regular users (76), or reporting socially desirable answers (77). That said, past research supports the accuracy of self-reported cannabis use as equivalent to biological measures such as urine tests (78). Third, the modest sample size and exclusive recruitment from MTurk users in the United States may result in lower generalization of results. MTurk is nevertheless the largest method of online crowdsourcing (79), and provides researchers access to hard-to-reach populations such as non-treatment seeking cannabis users (80). To further support the findings of this paper, future studies would benefit from recruiting regular users across different recruitment platforms, and over multiple time points to detect changes in mental health functioning, levels of cannabis use, and reckless behavioral patterns.

In conclusion, the present study has demonstrated that young adults who use cannabis on a regular basis are not a homogenous sample. The High Motives and Expectancies class experienced higher symptoms of psychosis, depression, anxiety, and problematic cannabis use, and were more likely to engage in reckless behavior such as attending work high or driving under the influence of cannabis. Understanding how these patterns of use are associated with poorer psychological functioning can help inform treatment design, utilizing a more person-centered approach. Future work should also build on these findings to examine whether patterns of regular use vary over time, and whether recovery is more effective with targeted interventions. Our findings also support the call to action for future studies to move away from focusing on only comparing regular users to occasional and non-users. As more jurisdictions continue to decriminalize cannabis for medicinal and recreational purposes, it is imperative that we understand the factors which place young adults at increased risk of harm.

## Data Availability Statement

The datasets presented in this study can be found in online repositories. The names of the repository/repositories and accession number(s) can be found at: https://osf.io/742hp/.

## Ethics Statement

The studies involving human participants were reviewed and approved by Monash University Human Research Ethics Committee (ethical approval number: CF15/1235–2015000576). The patients/participants provided their written informed consent to participate in this study.

## Author Contributions

DA, GY, and MY planned and developed the study protocol. DA collected the data. DA, GY, and LH analyzed data. DA, GY, LH, VL, LP, NS, and MY interpreted the results and wrote the manuscript. All authors contributed to the article and approved the submitted version.

## Conflict of Interest

The authors declare that the research was conducted in the absence of any commercial or financial relationships that could be construed as a potential conflict of interest.
