# Supplementary material for: Young Adults With Higher Motives and Expectancies of Regular Cannabis Use Show Poorer Psychosocial Functioning
Source: Front Psychiatry. 2020 Dec 15;11:599365. doi: 10.3389/fpsyt.2020.599365 (PMC7771276; doi:10.3389/fpsyt.2020.599365)
Supplement: Supplementary file 1 [file Data_Sheet_1.docx]

Supplementary Material

# Supplementary Figures and Tables

| Supplementary Table 1. Means and confidence intervals of demographic outcome variables (3-class model)^a^. | | | | |
| --- | --- | --- | --- | --- |
| Variable | Class 1  *M* (95% CI) | Class 2  *M* (95% CI) | Class 3  *M* (95% CI) | Significant Contrasts |
| Age | 26.53 (25.97, 27.1) | 25.7 (25.23, 26.16) | 25.22 (24.26, 26.19) | C1>C2*, C3* |
| Percentage of males | 56% (47%, 64%) | 61% (54%, 68%) | 66% (51%, 80%) |  |
| Percentage in a relationship | 51% (43%, 59%) | 38% (32%, 44%) | 47% (35%, 60%) | C1>C2* |
| Percentage who have completed secondary/high school | 79% (72%, 87%) | 74% (68%, 81%) | 75% (61%, 88%) |  |
| Percentage who are currently employed | 74% (66%, 82%) | 80% (74%, 86%) | 82% (70%, 93%) |  |
| Percentage who have Caucasian ethnicity | 74% (66%, 82%) | 79% (73%, 85%) | 75% (62%, 89%) |  |
| Percentage who live alone | 28% (21%, 36%) | 26% (21%, 31%) | 23% (12%, 35%) |  |
| *Note.* **p* < .05, ***p* < .01, ****p* < .001 ^a^Analyses were adjusted for age, gender, education, employment, Caucasian ethnicity, and total scores of the AUDIT, DAST and FTND. | | | | |

| Supplementary Table 2. Means and confidence intervals of cannabis use variables (3-class model)^a^. | | | | |
| --- | --- | --- | --- | --- |
| Variable | Class 1  *M* (95% CI) | Class 2  *M* (95% CI) | Class 3  *M* (95% CI) | Significant Contrasts |
| Age of first use | 17.12 (16.63, 17.61) | 16.28 (15.88, 16.69) | 16.44 (15.6, 17.28) | C1>C2* |
| Age of regular use | 20.47 (19.92, 21.03) | 19.35 (18.89, 19.8) | 19.23 (18.28, 20.18) | C1>C2**,C3* |
| Self-reported “high” during use (10 = very blazed) | 5.3 (4.98, 5.62) | 6.38 (6.11, 6.64) | 6.25 (5.7, 6.81) | C1<C2***,C3** |
| Percentage who sometimes/always drive high | 16% (9%, 23%) | 32% (26%, 39%) | 30% (17%, 44%) | C1<C2** |
| Percentage who sometimes/often go to work high | 12% (6%, 18%) | 26% (20%, 33%) | 40% (25%, 55%) | C1<C2**,C3** |
| Percentage with half or more peers using cannabis | 38% (29%, 47%) | 72% (65%, 78%) | 74% (60%, 88%) | C1<C2***,C3*** |
| Percentage who use cannabis all day | 7% (2%, 12%) | 22% (16%, 28%) | 26% (12%, 40%) | C1<C2***,C3* |
| Percentage usually using cannabis and alcohol | 19% (12%, 26%) | 16% (10%, 21%) | 30% (17%, 43%) | C2<C3* |
| Number of days using cannabis per week |  |  |  |  |
| 1-2 times | 50% (41%, 58%) | 26% (19%, 33%) | 32% (18%, 46%) | C1>C2***,C3* |
| 3-5 times | 27% (18%, 35%) | 30% (23%, 36%) | 35% (20%, 49%) |  |
| 6-7 times | 24% (16%, 31%) | 44% (37%, 52%) | 33% (19%, 48%) | C1<C2*** |
| Preferred type of marijuana |  |  |  |  |
| Mostly dried heads | 51% (42%, 60%) | 42% (35%, 50%) | 55% (39%, 71%) |  |
| Mostly dried leaves | 22% (15%, 29%) | 19% (13%, 26%) | 20% (7%, 33%) |  |
| Sinsemilla | 17% (10%, 24%) | 34% (27%, 41%) | 21% (7%, 34%) | C1<C2** |
| I don't know | 10% (5%, 16%) | 4% (1%, 7%) | 5% (0%, 11%) |  |
| Preferred route of administration |  |  |  |  |
| Joint | 25% (17%, 32%) | 14% (9%, 20%) | 29% (14%, 44%) | C1>C2* |
| Pipe | 28% (19%, 36%) | 29% (22%, 36%) | 24% (11%, 38%) |  |
| Water Pipe / Bong | 11% (5%, 17%) | 13% (8%, 18%) | 11% (2%, 19%) |  |
| Blunt | 10% (5%, 15%) | 12% (7%, 17%) | 13% (2%, 23%) |  |
| Vaporizer | 3% (0%, 7%) | 9% (5%, 13%) | 7% (0%, 14%) |  |
| Other | 23% (15%, 31%) | 23% (17%, 29%) | 17% (5%, 28%) |  |
| Preferred company during use |  |  |  |  |
| Alone | 76% (68%, 83%) | 77% (71%, 84%) | 77% (64%, 89%) |  |
| Friends/partner | 72% (64%, 80%) | 92% (87%, 96%) | 80% (68%, 93%) | C1<C2*** |
| Family | 7% (2%, 11%) | 20% (14%, 26%) | 9% (0%, 18%) | C1<C2** |
| Others | 4% (0%, 7%) | 9% (5%, 13%) | 3% (0%, 8%) | C1<C2** |
| Preferred location during use |  |  |  |  |
| In public | 31% (23%, 40%) | 44% (37%, 51%) | 42% (27%, 57%) | C1<C2* |
| At home | 44% (81%, 93%) | 95% (92%, 98%) | 93% (86%, 100%) | C1<C2* |
| At friend’s house | 61% (52%, 70%) | 83% (77%, 89%) | 74% (60%, 88%) | C1<C2*** |
| *Note.* **p* < .05, ***p* < .01, ****p* < .001 ^a^Analyses were adjusted for age, gender, education, employment, Caucasian ethnicity, and total scores of the AUDIT, DAST and FTND. | | | | |

| Supplementary Table 3. Means and confidence intervals of mental health and substance use outcome variables (3-class model)^a^. | | | | |
| --- | --- | --- | --- | --- |
| Variable | Class 1  *M* (95% CI) | Class 2  *M* (95% CI) | Class 3  *M* (95% CI) | Significant Contrasts |
| **Mental health outcomes:** |  |  |  |  |
| Total Apathy Evaluation Scale score | 42.9 (41.68, 44.12) | 42.35 (41.35, 43.36) | 41.92 (39.84, 44.01) |  |
| CAPE Positive Psychotic Experiences subscale | 24.54 (23.38, 25.71) | 26.95 (25.99, 27.91) | 29.57 (27.57, 31.57) | C1<C2**, C3***; C2<C3** |
| CAPE Negative Psychotic Experiences subscale | 20.64 (19.37, 21.92) | 22.89 (21.84, 23.94) | 24.76 (22.57, 26.95) | C1<C2**, C3** |
| DASS-21 Depression subscale | 2.47 (1.77, 3.18) | 3.67 (3.09, 4.26) | 4.84 (3.63, 6.05) | C1<C2*, C3** |
| DASS-21 Anxiety subscale | 1.92 (1.39, 2.46) | 2.76 (2.32, 3.2) | 4.37 (3.46, 5.29) | C1<C2*, C3***; C2<C3** |
| DASS-21 Stress subscale | 2.73 (2.12, 3.34) | 4.18 (3.67, 4.69) | 5.12 (4.07, 6.18) | C1<C2**, C3*** |
| Percentage who have ever sought mental health treatment | 14% (7%, 20%) | 19% (14%, 25%) | 10% (1%, 19%) |  |
|  |  |  |  |  |
| **Problematic cannabis use:** |  |  |  |  |
| Total CUPIT score | 24.59 (22.78, 26.41) | 31.96 (30.46, 33.45) | 33.6 (30.5, 36.71) | C1<C2***, C3*** |
| CUPIT Impaired Control subscale | 22.41 (20.86, 23.97) | 28.66 (27.38, 29.94) | 29.11 (26.45, 31.78) | C1<C2***, C3*** |
| CUPIT Problems subscale | 2.18 (1.65, 2.71) | 3.29 (2.86, 3.73) | 4.49 (3.58, 5.4) | C1<C2**, C3***; C2<C3* |
| CUPIT Cut-off score ≥ 12 | NA^b^ | NA^b^ | NA^b^ |  |
| CUPIT Cut-off score ≥ 20 | 70% (61%, 78%) | 95% (92%, 98%) | 92% (83%, 100%) | C1<C2***, C3*** |
|  |  |  |  |  |
| **Substance use outcomes:** |  |  |  |  |
| Total AUDIT score | 10.81 (9.68, 11.94) | 12.61 (11.67, 13.55) | 13.26 (11.31, 15.21) | C1<C2*, C3* |
| Total DAST score | 1.05 (0.81, 1.29) | 1.39 (1.19, 1.59) | 1.35 (0.94, 1.77) | C1<C2* |
| Total FTND score | 0.82 (0.45, 1.2) | 1.29 (0.98, 1.6) | 0.66 (0.03, 1.3) |  |
| *Note.* **p* < .05, ***p* < .01, ****p* < .001 ^a^Analyses were adjusted for age, gender, education, employment, Caucasian ethnicity, and total scores of the AUDIT, DAST and FTND.  ^b^NA indicates model could not be estimated due to zero cell count. | | | | |

## Supplementary Figures


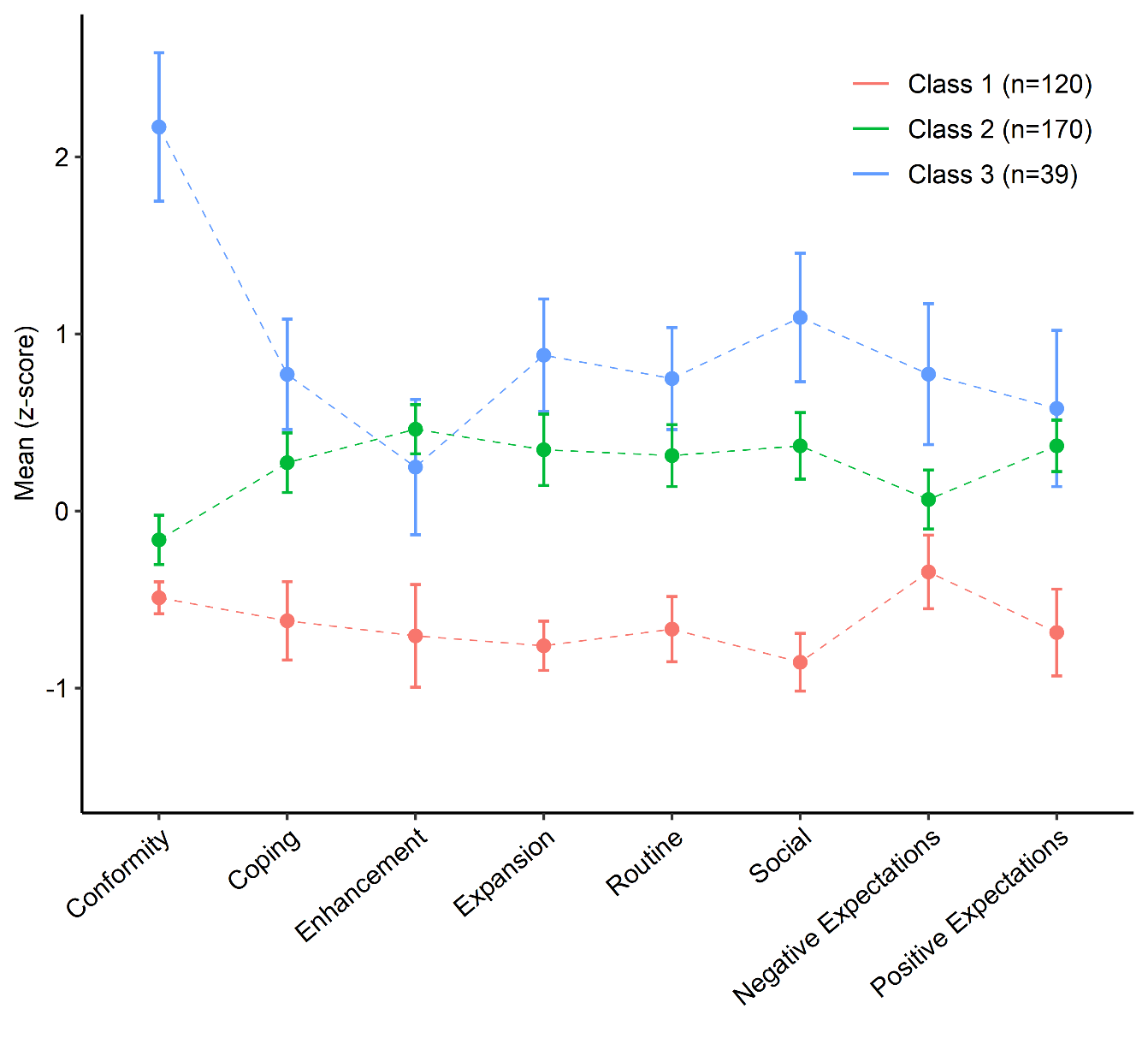


**Supplementary Figure 1.** Latent profile of participants based on marijuana use motives and cannabis use expectancies. Error bars represent 95% confidence intervals.
